# 非小细胞肺癌脑转移治疗的研究进展

**DOI:** 10.3779/j.issn.1009-3419.2020.102.39

**Published:** 2020-12-20

**Authors:** 雪 韩, 红梅 李

**Affiliations:** 1 266071 青岛，青岛大学青岛医学院 Qingdao Medical College, Qingdao University, Qingdao 266071, China; 2 266003 青岛，青岛大学附属医院肿瘤科 Department of Oncology, The Affiliated Hospital of Qingdao University, Qingdao 266003, China

**Keywords:** 肺肿瘤, 脑转移, 治疗, Lung neoplasms, Brain metastasis, Treatment

## Abstract

肺癌是全世界癌症相关死亡的首要原因，肺癌最常见的病理类型是非小细胞肺癌（non-small cell lung cancer, NSCLC），NSCLC引起的脑转移发病率一直呈上升趋势。脑转移严重影响患者的认知功能、生存时间及生活质量，预后极差，自然病程约1个月-3个月。经过治疗后中位生存时间也仅有3个月-6个月，1年生存率为14%，2年生存率仅为7.6%。脑转移的部位、数量、大小与其症状及生存期相关，有中枢神经系统症状的患者预后更差。脑转移瘤治疗的目标是优化总体生存率和生活质量，并优先保留神经认知功能。目前，NSCLC脑转移的主要治疗方式包括放疗、手术、化疗、分子靶向、免疫治疗。临床上要根据患者的异质性（临床特点、病理类型、组织分型等），对患者最佳的治疗方式进行多学科的评估。本文旨在对当前治疗方法的研究进展进行综述。

## Abstract (en)

Lung cancer is the leading cause of cancer-related deaths worldwide. The most common pathological type of lung cancer is non-small cell lung cancer (NSCLC). The incidence of brain metastases caused by NSCLC has been on the rise. Brain metastasis seriously affects the patient's cognitive function, survival time and quality of life, and the prognosis is extremely poor. The natural course of the disease is about 1 mon-3 mon. After treatment, the median survival time is only 3 mon-6 mon, the one year survival rate is 14%, and the two years survival rate is only 7.6%. The location, number, and size of brain metastases are related to their symptoms and survival. Patients with central nervous system symptoms have a worse prognosis. The goal of brain metastasis treatment is to optimize overall survival and quality of life, and to prioritize the preservation of neurocognitive functions. At present, the main treatments for brain metastases in NSCLC include radiotherapy, surgery, chemotherapy, molecular targeting, and immunotherapy. Clinically, it is necessary to conduct a multidisciplinary assessment of the best treatment methods for patients according to the heterogeneity of patients (clinical characteristics, pathological types, tissue types, *etc*). This article aims to review the research progress of current treatment methods.

肺癌是目前全球和中国发病率较高的恶性肿瘤之一，非小细胞肺癌（non-small cell lung cancer, NSCLC）是最常见（85%）的肺癌病理类型，脑是NSCLC远处转移的最常见部位。大约40%的NSCLC患者在病程中会发生脑转移^[1, 2]^。脑转移也是晚期NSCLC中致残和死亡的主要原因^[3]^。NSCLC脑转移的主要治疗方式包括放疗、手术、化疗、分子靶向、免疫治疗，可分为局部治疗和全身治疗两种类型。根据脑转移的症状、数量、大小和位置，可以采用立体定向放射治疗（stereotactic radiotherapy, SRT）、手术或全脑放射（whole brain radiation therapy, WBRT）对患者进行局部治疗^[4]^。对于那些无神经系统症状的脑转移，另一种选择是预先全身性治疗，包括靶向治疗或化疗^[4, 5]^。对于预后不良的患者，通常建议最佳支持治疗，包括类固醇治疗^[4]^。脑转移长期生存者很少，除了具有可靶向的驱动基因突变的患者，表皮生长因子受体（epidermal growth factor receptor, *EGFR*）和间变性淋巴瘤激酶（anaplastic lymphoma kinase, *ALK*）基因突变的脑转移患者中位生存期延长，对于脑转移瘤预后评分系统（graded prognostic assessment, GPA）最新的研究^[6]^是将*EGFR*和*ALK*基因状态合并入GPA系统，称为分子GPA（molGPA），molGPA预测更为准确，分数越高代表预后越好，更有助于临床决策。因此了解NSCLC脑转移的治疗方法的新进展，对延长NSCLC脑转移患者的生存期、改善患者生存质量和提高治疗效果意义十分重大，我们将在本综述中提供有关该主题的概述。

## 1 局部治疗

### 1.1 放疗

#### 1.1.1 全脑放疗

全脑放疗（whole brain radiation therapy, WBRT）一直被认为是脑转移患者的主要局部治疗方法之一，现已发现WBRT会对脑转移患者认知功能及生活质量造成不利影响，脑转移的放射治疗已从WBRT逐渐发展为各种放射策略[WBRT、手术+WBRT、立体定向放射治疗（stereotatic radiotherapy, SRT）+WBRT等]。WBRT适用于颅内有多发转移灶（转移灶≥3个，转移灶 < 3 cm）、体力状态（performance status, PS）评分不高的患者，也可用于立体定向外科（stereotactic radiosurgery, SRS）术后或脑转移瘤手术治疗后的辅助治疗。单独使用WBRT总缓解率可达到60%，6个月疾病控制率为50%，可以在1周-3周内使神经系统症状改善率达到70%-90%^[7]^。Quartz试验^[8]^比较了最佳支持治疗包括地塞米松联合WBRT（20 Gy/5 F）或仅地塞米松用于治疗预后较差的NSCLC患者。研究发现就改善生存率、提高总体生存质量方面或是减少类固醇的使用而言，WBRT不能为NSCLC脑转移患者提供临床获益。然而，大多数患者患有无法控制的胸部疾病（64%），颅外转移（54%）。WBRT在脑转移瘤治疗中的作用逐渐减弱，因为该技术与神经认知功能下降的风险有关^[9]^。为了既能提高脑转移控制率，又能保护患者神经认知功能，目前提出了智慧脑放疗（simultaneous modulated accelerated radiation therapy for elective brain, SMART-Brain）的概念，SMART-Brain是基于调强技术实施同步脑转移瘤加量照射和重点功能区域（如记忆功能区海马、听力区内耳等）保护的脑部放疗方法，患者在保护海马（≤10 Gy），保护内耳（≤15 Gy）的前提下行全脑放疗（30 Gy/10 F/2 wk）和脑转移瘤病灶（40 Gy-50 Gy/10 F/2 wk）高剂量放疗，已经在全国40多家中心开展多中心随机对照研究（CRTOG1702/1703）。对于寡转移患者，加用WBRT降低了脑转移的复发几率，但没有提高生存率; WBRT使神经功能损伤的风险明显增加; 部分预后好的有选择的患者有可能从WBRT中获益。对于多发脑转移患者，WBRT不一定能够延长其生存期和提高其生活质量; 对于一般状况差、颅外未控等患者，WBRT的选择需慎重。

#### 1.1.2 SRS

SRS有定位精确、剂量集中、损伤相对较小等优点，能够很好地保护周围正常组织，控制局部肿瘤进展，缓解神经系统症状，且对神经认知功能影响小，近年来已逐渐成为NSCLC脑转移的主要治疗手段之一。主要适用于单发、直径4 cm-5 cm以下、转移灶≤4个; WBRT后的挽救治疗; 单发脑转移术后辅助。中位总生存期（overall survival, OS）为9.3个月-10.8个月^[10]^，且治疗后不良反应少，而且经过SRS治疗的脑转移患者病情进展后可以再次接受SRS治疗，但SRS只是局部治疗，单独应用SRS治疗后，颅内、外病灶容易复发，因此主张联合WBRT治疗，积极控制颅外病灶，密切随访。有研究^[11]^通过对47例脑转移患者49个病灶手术后针对瘤床行SRS，1年、2年的瘤床控制率为86%、67%，显示出术后行SRS是安全有效的，尤其是对于直径≤3 cm的小肿瘤，可实现局部控制。JLGK0901研究^[12]^多中心前瞻性地观察了SRS用于多发性脑转移的价值，入组了1, 194例脑转移患者，按照转移灶的数目分为3组（1个、2个-4个、5个-10个），三组的OS分别为13.9个月、10.8个月、10.8个月，结果显示出转移灶数目在5个-10个的SRS疗效不差于2个-4个的疗效。SRS创伤小，副反应小，是5个-10个转移灶的另一选择。最新荟萃分析^[13]^显示SRS+WBRT联合治疗组降低了GPA < 2分和GPA≥2分的亚组中的脑肿瘤的复发，明显改善了脑部病灶的控制，未显示出比单独的SRS有明显生存获益，但是认知功能恶化发生率更高。一项单中心回顾性分析^[14]^脑转移瘤患者多程SRS，推迟WBRT的研究接受SRS两程以上的95例患者共652个转移灶，每例患者接受的SRS疗程中位数为2个（2个-14个），每个疗程SRS照射病灶中位数2个（1个-14个），从第一个和第二个SRS疗程开始的中位OS分别为18个月和11个月，结果显示对于初始SRS后发生远处脑转移的患者行多程SRS，推迟WBRT的方式是安全有效的。由于WBRT正面临质疑，SRS在脑转移治疗的地位中得到了很大提升，目前研究显示对于单发病灶，SRS与手术治疗的肿瘤控制率（tumor control probability, TCP）都高，生存疗效接近，考虑到SRS的非创伤性，优先选择SRS; 对于多发脑转移病灶，转移灶数目在5个-10个的SRS疗效不差于2个-4个的疗效。

### 1.2 手术治疗

外科手术治疗目前常用于肿瘤个数1个-3个、肿瘤大小 > 3 cm、肿瘤位置浅表、位于非重要功能区的部位病灶，并要考虑肿瘤的组织学类型及患者的全身状况等方面。由于大多数患者明确脑转移时已是晚期，所以手术治疗的选择应该谨慎^[6]^。对于病理不明确时、手术可以到达肿瘤部位时; 肿块较大、占位效应明显、伴有大片水肿者; 小脑部位较大转移灶（直径 > 2 cm）; 有应用激素不良或禁忌者更适于手术治疗。在一项回顾性研究^[15]^中比较了射波刀（cyberknife）治疗与外科手术在NSCLC孤立性脑转移中的治疗差异，观察终点为OS，治疗转移的局部控制率（local control of treated metastasis, LC）和颅内肿瘤控制率（intracranial control, IC）。结果显示在OS、LC和IC中，NSCLC的孤立性脑转移的射波刀治疗和外科手术之间无统计学差异。但是，射波刀的侵入性较小，可能更适合患者。EORTC 22952-26001研究^[16]^分析了手术或者SRS局部治疗后是否需要加用WBRT，该研究共入组了具有稳定的全身性疾病或无症状原发性肿瘤且PS为0分-2分的实体瘤（不包括小细胞肺癌）的1个-3个脑转移瘤的359例患者，手术或SRS后随机分为接受WBRT（30 Gy/10 F）或观察组，其中共266例患者可以获得GPA数据，在GPA 0-2.5组和2.5-4.0组有无WBRT均对OS无明显影响（*P* < 0.99, *P*=0.87）。目前对于手术或者SRS局部治疗后是否需要加用WBRT是争论的热点，理由如下：WBRT会导致不可逆的神经认知功能的损伤，但局部治疗失败后挽救性治疗仍然有效。目前的临床研究显示相较于单纯的手术或SRS，WBRT能显著提高脑转移病灶的控制率（病灶数目4个以下），只显著提高了单个脑转移病灶患者的生存疗效。手术治疗在NSCLC脑转移患者中应用有限。

## 2 全身治疗

### 2.1 化疗

脑转移患者的化疗选择受到肿瘤抵抗、无效药物和血脑屏障（blood-brain barrier, BBB）的限制，但化疗仍是NSCLC脑转移重要的综合治疗方法之一。以顺铂、卡铂为主的铂类药物为基础，联合第三代细胞毒类药物可给NSCLC脑转移患者带来生存获益^[6]^。一项在WBRT后一线接受高剂量培美曲塞联合顺铂维持治疗的NSCLC脑转移患者的疗效和安全性的研究结果^[17]^显示，颅内客观控制率为68.8%，脑转移的中位无进展生存期（progression-free survival, PFS）为13.6个月，中位OS为19.1个月。有证据表明对于无症状的脑转移患者，培美曲塞-铂方案的前期全身疗法可能是这些患者的合理选择^[18]^。一项病例报告^[19]^显示：培美曲塞联合顺铂治疗无症状的肺腺癌脑转移患者，经2周期治疗后观察到肺部病灶及脑转移病灶均取得了部分缓解的效果。替莫唑胺（Temozolomide, TMZ）是一种口服的、能透过BBB的烷化剂，对NSCLC脑转移的控制有较好的疗效（78%）。TMZ同步WBRT能显著提高脑转移的控制率。一项比较脑放疗联合TMZ与单独脑放疗作为脑转移患者一线治疗疗效的荟萃分析^[20]^显示：放疗联合TMZ可以显著提高脑转移患者的客观缓解率（objective response rate, ORR）。

### 2.2 分子靶向治疗

由于很难从脑转移患者中获得组织标本，限制了其诊断和分子表型的明确，影响了治疗方法的发展。对血浆中无细胞肿瘤DNA的分析（考虑进行液体活检）有助于表征颅外肿瘤。但是，血浆中无细胞的肿瘤DNA数量有限，可能无法可靠地捕获脑转移患者基因组变化情况。一项回顾性研究^[21]^通过调查30例肺腺癌脑转移患者*EGFR*基因状态，其中16例患者在脑脊液或肿瘤组织中检测到*EGFR*激活突变为阳性，而脑脊液中具有*EGFR*突变的大多数患者在EGFR酪氨酸激酶抑制剂（EGFR-tyrosine kinase inhibitors, EGFR-TKIs）的治疗下均取得了良好的反应。可见通过对脑脊液中无细胞的肿瘤DNA进行分析可提供实时精确获取和监测基因组信息并指导精确靶向治疗的机会。

#### 2.2.1 EGFR-TKIs

EGFR-TKIs是*EGFR*突变进展期NSCLC的一线标准治疗^[22]^。EGER-TKIs脂溶性好，能一定比例的透过BBB，已显示出在NSCLC脑转移患者中激活突变的有效性^[23]^，可用于*EGFR*突变的NSCLC脑转移患者治疗。目前在临床上广泛应用的第一代TKIs有吉非替尼（Gefitinib）、厄洛替尼（Erlotinib）及埃克替尼（Icotinib），二代TKIs有阿法替尼（Afatinib）、达可替尼（Dacomitinib），三代TKIs奥希替尼（Osimertinib, Tagrisso, AZD9291）。

近年来，对于EGFR-TKIs治疗NSCLC脑转移患者的临床效果逐渐受到了关注，多项临床试验证明其有效性。一项回顾性研究^[24]^比较了埃克替尼和吉非替尼对*EGFR*突变的NSCLC脑转移的疗效，两组之间的颅内ORR（66.6% *vs* 59.1%）和疾病控制率（disease control rate, DCR）（85.7% *vs* 81.8%）没有显著差异，颅内PFS（intracranial PFS, iPFS）中位数分别为8.4个月和10.6个月，显示出EGFR-TKIs治疗有效。另一项研究^[25]^显示厄洛替尼治疗的患者的中位OS并没有比吉非替尼治疗的患者的中位OS显著更长（25.0个月*vs* 18.1个月），但是在预防颅内病变和延长生存方面比吉非替尼更有效。

相较于第一代TKIs药物，第二代TKIs提高了*EGFR*突变的NSCLC患者的PFS与OS，并在脑转移患者中有效。在阿法替尼与化疗在*EGFR*突变的NSCLC患者的两项临床研究的效果比较中^[26]^，一项是阿法替尼或顺铂联合培美曲塞治疗*EGFR*突变（LUX-Lung 3）转移性肺腺癌患者的III期临床研究中，以及另一项比较了阿法替尼与铂类化疗在*EGFR*突变的IIIb期或IV期肺腺癌（LUX-Lung 6）患者中一线治疗随机、开放III期研究的疗效，结果显示对于有脑转移的患者，阿法替尼与化疗相比都有改善PFS的趋势（LUX-Lung 3：11.1个月*vs* 5.4个月; LUX-Lung 6：8.2个月*vs* 4.7个月）。在综合分析中，对于有脑转移的患者，阿法替尼与化疗相比，PFS显著改善（8.2个月*vs* 5.4个月）。可见与化疗相比，阿法替尼显著提高了脑转移患者的ORR。达可替尼与阿法替尼具有相似的安全性^[27]^。

第三代TKIs奥希替尼不可逆抑制*EGFR*基因敏感突变和T790M突变的肺癌细胞，在脑组织中分布较前两代TKIs更高，穿透BBB的能力更强，PFS已超过18个月^[28]^，可延长PFS并提高ORR。III期FLAURA研究^[29]^显示与吉非替尼或厄洛替尼相比，使用奥希替尼可降低中枢神经系统（central nerval system, CNS）进展的风险。在AURA3研究^[30]^中奥希替尼与铂类联合培美曲塞在T790M阳性晚期NSCLC中CNS的中位PFS分别为11.7个月和5.6个月，表现出优于化疗的CNS疗效。

尽管用TKIs治疗*EGFR*突变型肿瘤改善了肿瘤控制，但所有肿瘤最终都将产生耐药性，已经报道了T790M突变是几种耐药途径的主要机制^[31]^。一、二代EGFR-TKIs治疗进展后，T790M突变的阳性率达50%以上。NSCLC患者的软脑膜病变（leptomeningeal lesions, LMD）发生率为3%-5%^[32]^，对于逐渐进展的LMD，考虑奥希替尼（不管T790M状态）或厄洛替尼冲击疗法。对于疾病进展，应考虑重复组织活检。疾病进展可考虑使用奥希替尼治疗T790M突变的患者，而仅CNS疾病进展的患者可考虑采用放射疗法（SRS和/或WBRT）和提高厄洛替尼剂量。

Icotinib Brain研究^[33]^（NCT01724801）通过入组*EGFR*突变型且未经EGFR-TKIs治疗的伴有脑转移（脑转移灶≥3个）的晚期NSCLC患者，比较埃克替尼与WBRT（30 Gy/10F）±化疗的疗效差异，两组的中位iPFS分别为10.0个月和4.8个月。结果显示埃克替尼可提高携带EGFR突变的NSCLC脑转移患者的iPFS和PFS，优于全脑放疗±化疗，可获得更好的ORR，埃克替尼应该用于携带*EGFR*突变的脑转移NSCLC患者的一线治疗，但该试验存在一定的局限性，放疗采用的是疗效较差的WBRT，而不是尽可能采用SRS; 80%以上是无症状脑转移; 两组均是在疾病进展后才采取另一种治疗，没有考虑两者的有机联合。EGFR-TKIs联合放疗具有协同作用，药代动力学分析^[34]^显示，WBRT联用吉非替尼可增强吉非替尼渗透至脑脊液内的能力，增加吉非替尼在脑脊液内的浓度。对于真实世界而言，EGFR-TKIs联合放疗是*EGFR*突变型患者的标准治疗。一项研究^[35]^显示对于*EGFR*突变的NSCLC脑转移患者，先用SRS与先用靶向治疗的中位OS分别为34.1个月*vs* 19.4个月（*P*=0.01），亚组分析显示，先用SRS与先用靶向治疗的中位OS为58.4个月*vs* 19.4个月（*P*=0.01），而先用WBRT与先用靶向治疗的中位OS为29.9个月*vs* 19.4个月（*P*=0.09）。耶鲁大学多中心数据分析（ASTRO2016）入组2008年-2014年4个中心共162例*EGFR*敏感突变NSCLC脑转移患者，先用放疗和先用EGFR-TKIs进展后再放疗患者的OS分别为29.4个月*vs* 20.5个月（*P*=0.001, 5），亚组分析显示，先用SRS和先用EGFR-TKIs组患者OS为40.8个月*vs* 20.5个月（*P*=0.000, 1），先用WBRT和先用EGFR-TKIs的OS为25.3个月*vs* 20.5个月（*P*=0.07）。对于*EGFR*敏感突变的NSCLC脑转移患者而言，靶向治疗可以取得很好的疗效，靶向治疗与放疗的联合应用有可能能够提高部分患者的疗效，先用SRS较先用EGFR-TKIs的PFS与OS均显著延长，WBRT与EGFR-TKIs进展后再行挽救性放疗效果差异不大，但最佳联合方式有待于进一步开展大样本前瞻性研究确定。

#### 2.2.2 ALK-TKIs

*ALK*融合基因是NSCLC另一个治疗靶点，包括一代药物克唑替尼（Crizotinib），二代药物阿来替尼（Alectinib）、布加替尼（Brigatinib）、塞瑞替尼（Ceritinib），三代药物劳拉替尼（Lorlatinib）。

第一代ALK抑制剂克唑替尼与培美曲塞联合铂类化疗相比，克唑替尼对*ALK*+ NSCLC脑转移患者颅内转移瘤控制率更高^[7]^，但其穿过BBB的能力较低，大多数于1年左右发生耐药，治疗的大多数患者最终仍会经历CNS的进展。相关研究^[36]^显示与克唑替尼相比，阿来替尼可以预防或延迟脑转移的发生。III期ALEX研究^[37]^显示对于先前未经治疗的晚期*ALK*+ NSCLC的患者，无论先前是否有CNS疾病或放疗，与克唑替尼相比，阿来替尼具有优越的CNS活性，并显著延迟了CNS的进展。在NCT01449461和II期随机试验ALTA^[38]^中显示了布加替尼在克唑替尼治疗过的*ALK*+ NSCLC产生显著的颅内反应和持久的iPFS。阿来替尼和布加替尼在未经治疗的*ALK*+ NSCLC中优于以前的标准克唑替尼，PFS通常会超过2年-3年^[28]^。多项研究^[39-41]^显示作为高效高选择性二代ALK-TKIs塞瑞替尼，对于在克唑替尼治疗失败后的*ALK*+ NSCLC患者中获得显著的临床收益，并且是一种比化疗更有效的治疗选择。一项全球性II期研究^[42]^显示与劳拉替尼广泛的*ALK*突变覆盖率和CNS渗透性一致，劳拉替尼在未接受过治疗的*ALK*+ NSCLC患者和克唑替尼、二代ALK-TKIs治疗进展的患者中均表现出显著的整体和颅内活性。因此对于*ALK*+ NSCLC脑转移患者可以在治疗的各个阶段应用广泛认可的ALK-TKIs治疗，临床工作的主要挑战是寻找可为患者带来最佳生存获益的ALK-TKIs的序列和组合。

### 2.3 抗血管生成药物

血管生成主要由血管内皮生长因子（vascular endothelial growth factor, VEGF）途径介导，对于原发性和转移性脑病变中的肿瘤存活、生长和侵袭至关重要。贝伐珠单抗（Bevizumab）是一种重组人源化单克隆抗体，可通过空间阻断其与VEGF受体的结合来中和VEGF的生物学活性。贝伐珠单抗联合化疗是中国晚期肺癌患者一线标准治疗。一项回顾性分析^[43]^提供了支持性证据，与单纯化疗相比，贝伐珠单抗联合化疗可以显著降低晚期NSCLC患者的脑转移发生率。研究^[44]^发现对于NSCLC有症状脑转移的患者基于贝伐珠单抗的化疗耐受性好且有效：PFS为9.1个月，OS为9.6个月。贝伐珠单抗在血源性脑转移模型中^[45]^可抑制已建立的NSCLC脑转移的生长。然而由于担心脑出血和血栓栓塞性疾病的风险更大，常将具有CNS转移的患者排除在抗血管生成药物的试验之外。因此，尚不清楚抗血管生成药物在NSCLC脑转移患者中的整体疗效和安全性。但是动物模型结果显示出含贝伐珠单抗的方案对于NSCLC脑转移患者可能是一种有前途的治疗选择。

### 2.4 免疫治疗

免疫检查点抑制剂（immune checkpoint inhibitors, ICI）现在被迅速用于治疗晚期NSCLC，特别适用于表达程序性死亡受体配体1（programmed cell death ligand 1, PD-L1）蛋白的肿瘤。ICI作为单一疗法以及与化疗结合已成为晚期NSCLC患者一线治疗策略中的治疗标准^[46]^。尽管脑转移发生率很高，但未经治疗和/或不稳定脑转移的患者仍被排除在关键性ICI NSCLC试验中^[47-57]^。排除这些患者是由于ICI的大小，ICI可能无法穿过血脑肿瘤屏障（blood-brain barrier around the tumor, BTB）。迄今为止，已经批准了5种药物用于肺癌脑转移患者：抗PD-1药物派姆单抗（Pembrolizumab）和纳武单抗（Nivolumab）以及抗PD-L1药物德瓦鲁单抗（Durvalumab）、阿特珠单抗（Atezolizumab）和Avelumab。PD-1抗体（Pembrolizumab）单一疗法在NSCLC脑转移患者中显示出20%-30%的脑反应率^[58]^。CheckMate017/057/063研究共纳入971例患者，分为纳武单抗组544例（伴脑转移46例）和多西他赛组427例（伴脑转移42例），脑转移入组条件为无症状、稳定的脑转移且经过治疗、入组前超过2周无神经系统症状，两组的中位OS分别为8.4个月、6.2个月，结果显示对于伴有脑转移的患者，纳武单抗相比多西他赛显示出总生存获益倾向。一项回顾性队列研究^[59]^共纳入54例非鳞NSCLC患者，分为卡铂/培美曲塞组37例（脑转移患者12例）和卡铂/培美曲塞+派姆单抗17例（脑转移患者6例），脑转移患者无症状且至少有1个可测量病灶，对于整组人群两组的ORR分别为40.5% *vs* 53.3%，对于脑转移患者两组的ORR分别为58.3% *vs* 80%，结果显示派姆单抗联合化疗方案较单纯化疗可增加患者ORR，在脑转移的患者中得到同样结论。2019年美国临床肿瘤学会（American Society of Clinical Oncology, ASCO）会议上回顾性分析国家癌症数据库（National Cancer Database, NCDB）探索增加免疫治疗对接受颅内放疗的NSCLC脑转移患者的生存影响，共纳入13, 998例NSCLC患者（545例患者接受免疫治疗，13, 545例患者未接受免疫治疗），结果显示接受免疫治疗的患者中位OS为13.1个月，未接受免疫治疗的患者中位OS为9.7个月，接受免疫治疗是NSCLC颅内转移患者总生存率增加的独立预测因子。SRT后立即给予纳武单抗治疗耐受性良好^[60]^，一项回顾性研究显示放疗联合ICI治疗晚期NSCLC脑转移患者，对于放疗 < 3个月者颅内控制率更好，提示放疗联合免疫治疗的时间窗值得进一步探究。美国莫菲特癌症中心回顾性研究^[61]^了17例NSCLC（共49处脑转移病灶）接受免疫治疗联合CNS放疗，22处（45%）病灶为放疗后序贯ICI治疗，13处（27%）为放疗同步ICI治疗，结果显示免疫治疗联合放疗，尤其是免疫治疗前或同步接受SRS，可显著提高CNS控制率（57%）。霍普金斯医院回顾性分析2010年-2016年间所有接受SRS-SRT治疗伴有脑转移的NSCLC、黑色素瘤及肾癌患者^[62]^，评估不同治疗方式的安全性和有效性，提示免疫治疗与SRT同步更能改善脑转移患者的OS且降低颅内新发病灶的发生率。免疫联合放疗总体安全性好，但是相关研究^[63]^显示免疫联合放疗增加有症状放射性脑坏死风险（20% *vs* 6.7%）。脑转移患者治疗方向多元化发展，免疫联合化疗、免疫联合放疗均显示出较好的临床获益，但联合治疗的获益人群，联合的时机以及不良反应的预测及处理更加值得关注。

综上所述，NSCLC脑转移患者的预后极差，严重影响患者的生存及生活质量。随着晚期NSCLC的治疗选择数量的增加，一线治疗的最佳选择将取决于患者因素，例如脑转移瘤的存在、*EGFR*突变的类型、患者耐受性以及长期的后续治疗的选择。对于寡转移患者，加用WBRT降低了脑转移的复发，但没有提高生存率; WBRT使神经功能损伤的风险明显增加; 部分预后好的有选择的患者有可能能从WBRT中获益。对于多发脑转移患者，WBRT不一定能够延长其生存期和提高其生活质量; 对于一般状况差、颅外未控等患者，WBRT的选择需慎重。由于WBRT正面临质疑，SRS在脑转移治疗的地位中得到了很大提升，目前研究显示对于单发病灶，SRS与手术治疗的TCP都高，生存疗效接近，考虑到SRS的非创伤性，优先选择SRS; 对于多发脑转移病灶，转移灶数目在5个-10个的SRS疗效不差于2个-4个的疗效。手术治疗在NSCLC脑转移患者中应用有限。对于可靶向的敏感基因突变的NSCLC脑转移患者而言，靶向治疗可以取得很好的疗效，靶向治疗与放射治疗的联合应用有可能能够提高部分患者的疗效，先用SRS较先用靶向治疗的PFS与OS均显著延长，WBRT与靶向治疗进展后再行挽救性放疗效果差异不大，但最佳联合方式有待于进一步开展大样本前瞻性研究确定。目前尚不清楚抗血管生成药物在NSCLC脑转移患者中的整体疗效和安全性。但是动物模型结果显示出含贝伐珠单抗的方案对于NSCLC脑转移患者可能是一种有前途的治疗选择，未来还需要大样本前瞻性研究来明确。免疫治疗为NSCLC脑转移患者提供新希望，但免疫治疗的时机和方式有待未来更多探索。在目前实际的临床工作中要注意排兵布阵，正所谓“有靶打靶，无靶免疫”，一定要合理优化各种治疗方式，争取最好的治疗效果，优先保留NSCLC脑转移患者的神经认知功能，使NSCLC脑转移患者获得最大的生存收益及最佳的生存质量。
